# Nurse Practitioner Mental Health Care in the Primary Context: A Californian Case Study

**DOI:** 10.3390/healthcare3010162

**Published:** 2015-03-18

**Authors:** Theane Theophilos, Roger Green, Andrew Cashin

**Affiliations:** 1 School of Health and Human Sciences, Southern Cross University, PO Box 157, Lismore, NSW 2480, Australia; E-Mail: theane.theophilos@scu.edu.au; 2 College of Health, Human Services, and Nursing, California State University Dominguez Hills, 1000 East Victoria Street, Carson, CA 90747, USA; E-Mail: rgreen@csudh.edu

**Keywords:** primary care nurse practitioner, nurse practitioner curriculum, family nurse practitioner, primary care mental health

## Abstract

In America, mental health needs surpass the availability of specialized providers. This vulnerable population also has other obstacles for comprehensive care including gaps in medical coverage, stigma, economic barriers, and a geographical mal-distribution of qualified mental health professionals. A wide availability of primary care providers, including primary care and family nurse practitioners, are well-positioned to deliver integrated mental and physical health care. A case study from a Southern California Coachella Valley primary care clinic with integrated services is used to demonstrate the much-needed approach of care to address health disparities that face low-income immigrants, migrant workers, and others without access to specialized care centers and providers. It is argued that mental health care should be part of all holistic treatment provided by primary care and family nurse practitioners. This has implications for curricula and practice development.

## 1. Background

Internationally, it is recognized that “there is no health without mental health” [1]. In the USA, President Obama, in his address at the 2013 National Conference on Mental Health, asserted the now oft cited quote, “too many Americans who struggle with mental illness suffer in silence rather than seek help” [2]. Mental health disorders, of which depression and anxiety [2] are the two most common disorders, affect approximately one-quarter of the general population [1]. While fairly prevalent, less than half those affected choose to seek treatment. However, for those who do, there are many obstacles including stigma of such a disorder, economic disadvantages, gaps in insurance coverage, and limited availability of specialists. One viable solution to overcoming the obstacles includes broader utilization of primary care providers (PCPs), including family nurse practitioners. PCPs are, if consciously prepared through curricula and supervised practice, fully competent to diagnose, assess, treat, and evaluate individuals with mental disorders. The Coachella Valley is used to demonstrate possibilities through utilization of a case study.

## 2. Coachella Valley California

According to the U.S. Government, 43.7 million individuals experienced a mental health related issue in 2012 [3]. California, one of the largest states in the nation, reported nearly 1.8 million individuals with a mental health related issue [4].

The size of California warrants dividing the state into nine regions, namely: Central Coast, Greater Bay area, Inland Empire, Los Angeles County, Northern and Sierra, Orange County, Sacramento area, San Diego area and San Joaquin valley. The Coachella Valley is located in Riverside County, Southern California and comprises part of the Inland Empire and is in reasonable proximity to the Mexican Border. Given the seasonal agricultural nature of the region, many migrant families temporarily farm in the valley. Riverside County was significantly impacted during the recent economic recession, with above-average employment loss and foreclosures. Compounding the vulnerability of the region is the lack of mental health professionals. Presently, there are no training programs within the region dedicated to preparing such professionals.

## 3. The Need for a Primary Care Based Mental Health Service

The recent (2013) Health Assessment Resource Centre report on more than 350,000 residents from the Coachella Valley affirmed that one quarter (25.3%) of the residents reported a disorder of mental health [5]; additionally, an unfavorable, upward trend was noted given that 18% reported a mental health disorder in 2010. The most common mental health disorders in descending order were depressive disorder, generalized anxiety disorder and phobias, with phobias reported significantly more frequently (4.7%) than the 2010 sample (1.8%). Suicidal ideation remained consistent with previous years with a prevalence of 2.5%. Despite 78% of the Coachella Valley population reporting that they knew where to seek appropriate mental health treatment, only 25.3% of those in need reported actually seeking treatment [5].

When the Coachella Valley population was stratified by ethnicity, Hispanics/Latino adults were significantly less likely to have been treated with medication (83% not treated) for a mental health disorder in the past year than their white counterparts (53% not treated). The research does not comment on whether medication was in fact appropriate or desirable but it does suggest a possibility of inequitable access to specialist mental health services along racial/socio-economic lines. This data is in accordance with research conducted by Cook *et al.* (2014) in the USA, where nearly 7000 mental disorder episodes were analyzed, which showed that Latinos and Black participants were less likely to initiate and receive adequate mental health care [6]. Interestingly, Latinos had a significantly greater number of primary healthcare visits compared to other ethnic groups which means that access to quality mental health care in the primary care context is essential for this population [6].

It was noted that there was a disparity between mental health insurance coverage for Hispanic adults (28%) as opposed to white (58%) participants [5]. This finding was likewise reflected in literature [6]. Similarly, lower income brackets were significantly less likely to have mental health insurance coverage than those in the higher income brackets, with over 70% of those earning less than $25,000 USD/annum reporting no mental health insurance. This represents over 103,700 people with no coverage [5].

## 4. Co-location of Specialist Mental Health Clinicians with Primary Care Clinicians

One approach to promote access to specialist mental health services is co-location with primary care services. Nardi (2011) reported the benefits of the Health and Wellness Centre in Illinois, a nurse-managed primary health care center which implemented integrated mental and physical health care, which she helped initiate [7]. Each patient was screened for depression on initial consultation and onsite counselling, psychotherapy, psychiatric assessments, prescriptive services and consultations are available by designated mental health providers [7]. Other authors have advocated the same model of co-location of mental health services within the primary care setting [8,9,10,11]. However, this is predicated on availability of the specialist psychiatric service providers (including nurse practitioners), insurance coverage of patients and having primary care providers with adequate training and skills.

There are limited psychiatric nurse practitioners and psychiatrists in the Coachella Valley. There are some rural areas of the Coachella Valley, especially in the Eastern Region, where there are no psychiatric services due to the remote location. Patients typically need to travel closer to the three hospitals in the region for psychiatric services. There is one crisis center operated by Riverside County in the city of Indio that provides emergency assessments and crisis stabilization. If psychiatric hospitalization is required patients are typically transferred to the county hospital located approximately 109 km away. There are some private hospitals that offer inpatient psychiatric services with the closest being approximately 85 km away.

## 5. Integrating Mental Health Services as Part of Primary Care

Another approach to service delivery is general primary care nurse practitioners and family nurse practitioners undertaking the mental health screening, treatment and referral to specialist services where necessary. Literature elucidates that around one third of primary care physician time is consumed with mental health issues [12]. Yet, currently there is a well-documented physician shortage in North America, especially in the primary care and mental health/psychiatry arena [13,14,15].

Thus, it makes sense that mental health care becomes a routine part of primary care delivered by all clinicians. In a Delphi study conducted by McIlrath *et al.* (2010) almost 90% of the primary care expert panel comprising psychiatrists, general medical practitioners, nurse practitioners, practice nurses, mental health nurses and health visitors (a role unique to the UK) surveyed asserted that nurses should view depression care as a routine part of their primary care role [16]. Saur *et al.*’s (2007) survey of patient perceptions likewise reinforced that it is the preference of health consumers to receive their psychiatric primary care in conjunction with other primary care services [17]. Thus there is a need for primary care nurse practitioners and family nurse practitioners to provide treatment for common mental health disorders.

We know that nurse practitioners are in fact already undertaking mental health services. Hanrahan and Sullivan-Marx’s (2005) cross-sectional analysis of over 700,000 Medicare claims in the US presented an interesting insight into mental health service provision [18]. The data showed that Advanced Practice Nurses (which previously have comprised both nurse practitioners and clinical nurse specialists) when compared with other primary care providers including primary care physicians, social workers and psychologists, were more likely to service older clients, in more rural locations, and clients who qualified for the poverty threshold. However, there is a paucity of published literature that describes the services provided by primary care nurse practitioners and family nurse practitioners as separated from specialist psychiatric or mental health nurse practitioners.

## 6. Person Centered Care as Central to Nurse Practitioner Philosophy

Primary care nurse practitioners and family nurse practitioners’ practice is based on a holistic care framework in which the person is the center of care. This is reflected in the National Organization of Nurse Practitioner Faculties’ (2012) core competencies which stipulate that each practitioner:

“*provide patient-centered care recognizing cultural diversity and the patient or designee as a full partner in decision-making; works to establish a relationship with the patient characterized by mutual respect, empathy, and collaboration; creates a climate of patient-centered care to include confidentiality, privacy, comfort, emotional support, mutual trust, and respect; incorporates the patient’s cultural and spiritual preferences, values, and beliefs into health care; and preserves the patient’s control over decision making by negotiating a mutually acceptable plan of care*”.

[19]

There is much discussion in the literature about holistic, integrated, consumer-focused care where health consumers are assumed to be experts on their own values, preferences and treatment goals [7,11,20,21,22,23,24].

Zolnierek (2014) asserted that “knowing the patient” was central to all quintessential nursing care [25]. This knowledge is based on three central themes: care, relationship and expert practice. Essentially these equate to the patient feeling cared for and connected to the nurse. Zolnierek (2014) related this deep knowledge to sound clinical judgement, where the practitioner is cognizant of the typical patient responses and engaged with these concerns.

This “knowing” may be considered as one of the components to “person centered care” which is synonymous with patient-centered care discussed above. Person-centered care is promoted as the gold-standard model of care for patients, especially certain sub-groups including dementia patients [24,26]. Person-centered care places the person at the center of care considerations where behaviours and symptoms are understood from the patient’s perspective, it likewise aims to create a positive psychosocial environment where relationship and communication are prioritized [26]. It is reported in health literature that there is a correlation between high person-centered care and increased engagement and ability in daily activities and higher reported patient well-being [26,27,28].

A study undertaken by Green *et al.* (2013) measured the degree of disclosure of concerns by wound patients. Interestingly, on 40% of occasions patients failed to discuss their concerns with their treating nurse [29]. Of even greater note was that, of the patients who did disclose to their nurse, 8% had their concerns overlooked and a further 30% who did discuss their concerns did not have them acted upon [29]. This study is evidence of the need for effective person centered models to be included as part of the curriculum and employed in clinical practice.

Time and motivation, have likewise been identified as barriers to primary care. Numerous studies have cited that a restriction in nurse time, especially in the context of practice environments, can negatively impede provision of person-centered care [25]. This lack of nurse practitioner time may be due to greater work caseload demands, increasing consumer expectations and an expanding scope of practice [30]. Provider motivation was also cited as a potential barrier to person centered care and thus, as part of building capability, nurse practitioners need to focus on knowledge of and attitude to person centered care. We advocate that it should be taught in didactic education and modelled and mentored in practice [28].

Person centered care is totally congruous with the recovery focus advocated by specialist mental health nurses. The client-centered paradigm has been inherent in mental health nursing rhetoric since its clinical conceptualisation in the 1950s and 60s, when the three facilitative conditions for the clinician were described as “warmth, genuineness and empathy” [31]. This notion is more widely discussed in mental health literature today in terms of the recovery model, a model which builds upon the foundation of person centered care, but according to Gavan (2011) takes a more optimistic approach with the addition of the idea of recovery [32]. The notion of hope is central to the recovery model, with a focus on “recovery to”: that is, what may lie beyond the illness, not just the traditional “recovery from” model which has its focus on the biomedical illness that is being treated [32]. Davidson and Roe (2007) asserted that symptom amelioration is a valid focus, but not the only way to view the concept of recovery. They argued the right to self-determination and inclusion in community life despite the person continuing to suffer from mental illness is central [33]. This notion has very much been cradled within the mental health consumer survivor movement. In critiquing the person centered care model Gavan (2011) asserted that the recovery based model is superior in certain clinical contexts due to the empowering of consumer voice which emphasises reciprocity and interdependence, which is not necessarily implicit in the therapeutic relationship espoused within person centered care [32]. Gordon (2013) cautioned a more nuanced approach and advised against a dualistic “recovery from” as the outcome of concern *versus* “recovery in” as a process paradigm [34].

It is evident that the philosophical underpinning of specialist mental health care is consistent with the philosophical perspective of primary care and family nurse practitioners. The only blocks to delivery can then be knowledge born of tertiary curricula, attitude or policy inhibitors built within the funding model.

## 7. Curriculum

The need for appropriate education that equips nurse practitioners in primary care in mental health capabilities is evident. We know that all registered nurses have to undertake a mental health component as part of their comprehensive undergraduate curriculum in Australia and the US. However, do nurse practitioners advance on this in their curriculum? A comprehensive literature review was undertaken to determine primary care nurse practitioner’s capability and preparedness to undertake mental health assessment and intervention. The databases Pubmed, CINAHL, PSYCInfo and MEDLINE were searched using the key terms “curriculum”, “education”, “curriculum mapping”, “university”, “family nurse practitioner”, “nurse practitioner”, “primary care”, “nurs*”, “mental health”, “psych*”, “core capacities”, “capabilities”, “competence” and “scope”.

After this comprehensive review, no such studies were identified with regard to mental health curriculum components for the primary care nurse. Primary care nurse practitioner preparation, and consequently capability to meet mental health service needs, was not identified as an area of discussion or study in the literature. There is currently no identified published literature in peer reviewed journals on mental health components at the Masters or Doctoral level primary care/family nurse practitioner tertiary programs.

One suggestion in evaluating tertiary course syllabus, is to map curricula against core mental health standards developed. Musselwhite and Freshwater (2005) provided such an example with registered nurses where numerous mental health courses were mapped against “capable practitioner” standards developed in the United Kingdom [35]. No such work was identified with nurse practitioner courses in either specialist mental health programmes or primary care (family) programmes. Additionally, there was no data on the integration of person-centered care practice within the family or primary care practitioner pedagogic curricula.

The case study (Box 1) while not representing any novel approach to treatment, demonstrates the possibility afforded through promotion of access to capable mental health care.

Box 1 **Study of a clinical example**

Maria was a 55 year old Hispanic female who was a new patient to this clinic. She presented with complaints of persistent nausea, weight loss and fatigue. Onset of symptoms were gradual over the past 6 weeks. Presently, she described that it is difficult for her to feel good about going to work and that she had missed multiple days. She attributed her missing work to the fatigue and lack of interest. When questioned further, she estimated that her weight loss was about 10 pounds, but losing the weight had not been intentional. She described having irregular sleep patterns with difficulty with initially falling asleep and remaining asleep. When she awoke during the night, she reported that her mind was racing and she had worrisome repetitious thoughts regarding her current financial status and missing so much work. She could not participate in the state sponsored health plan due to her immigrant status.

Maria relayed that she had two adult daughters. Each daughter had one daughter. Maria lived in a multi-generational household with her spouse, her sister, the two daughters and the two grandchildren. Her husband was employed with a local landscape company and had work seasonally.

In the past she described having had higher “fats in her blood” and was on medication. She quit the medications due to cost. Maria stated that she had no alcohol or illicit drug use. She did not hear any voices unexpectedly, nor had she experienced any visual or other auditory hallucinations. Marie relayed that she had no thoughts of harming herself or others.

Physical Exam

Vital signs were stable and within expected parameters. Height is 5'4'' and weight was 200 pounds (BMI 35.4 = Obese)

Affect: Blunted but reactive in mood congruous manner, slow to speak in broken English. (Daughter was present to translate and support in the conversation as needed, and as requested by Maria)

Head, Eye, Ear, Nose and Throat Exam: unremarkable findings

CV: Normal exam with regular heart rate, clear heart sounds, no murmurs.

Lungs: Clear to auscultation, without wheezing or diminished breath sounds

Abdomen: soft, non-tender

Mental Status: Speech, normal rate, volume, and tone. Willingly engaged in eye contact. No hesitancy in speech (predominantly Spanish) or increased latency of response. Short term recall was grossly intact. Oriented to person, place, time. Concentration normal. Thought processes logical and sequential. No hallucinations or delusions. Mood—feeling “sad”. Rapport is established. Became tearful when discussing employment.

Zung depression score was 60 (normal > 75); indicating depression.

Diagnosis by family nurse practitioner. Major Depressive Episode (DSM V criteria met).

Plan: Maria was consulted as to her beliefs surrounding pharmaceutical intervention. She agreed to start medication and willingly signed a no harm contract. She was willing to work with social services to assist with enrolment in State health plan.

Sertraline HCL, 50 mg once nightly was initiated after a discussion of use and possible side effects (including effect on sleep).

Followed up in 2 weeks; No side effects experienced from medication. Sleep improved. Feeling more confident in attending work.

For social interaction and spiritual health, Maria suggested continuing attendance at her place of worship. She will add at least one more pleasurable social event per week, such as the healthy cooking classes offered at the local community centre. Maria was happy to try this and agreed to keep a diary to discuss at the next appointment scheduled in two weeks.

## 8. Conclusions

With limited access to specialist psychiatric/mental health nurse practitioners and psychiatrists in the Coachella Valley and a relatively stronger availability of primary care and family nurse practitioners in the valley, it is clear that services delivered by the latter need to involve a holistic approach that includes mental health care. The need is further accentuated by the demographic profile of the area and the related complexities of insurance coverage. Methods to support building confidence and competence in the domain of mental health care warrant further exploration. In this framework, a thorough psychiatric assessment is necessary. Given that nurse practitioners in primary care may be the only treatment provider available, treatment needs to move beyond screening to beginning intervention. Core capabilities in this domain need to be built through all nurse practitioner programmes and developed in practice through engagement with clinical supervision from specialist psychiatric nurse practitioners and/or other psychiatric specialists. There is a paucity of literature related to over-all US nurse practitioner curricula in terms of core mental health content. Core mental health capability building needs to be built into all curricula to ameliorate barriers to care present through knowledge level, motivation and confidence.
